# COMMENT ON: Metabolic bone training in the United Kingdom: is the future of bone health at risk of breaking?

**DOI:** 10.1093/rap/rkae070

**Published:** 2024-06-03

**Authors:** Alice Cole, Ali S Jawad

**Affiliations:** Department of Rheumatology, University College London Hospital, London, UK; William Harvey Institute, Queen Mary University of London, London, UK

Dear Editor, We share Hawarden et al. concern regarding training in metabolic bone disease (MBD) [1]. To address this important issue, we organised a 3-hour session in MBD in the regular training programme for rheumatology trainees in the North Thames Region in May 2023. It was attended by 23 rheumatology trainee doctors. A number of consultants and trainees from care of the elderly medicine and endocrinology also attended. The programme was designed to be interactive, clinical and practical, covering as many aspects of MBD (Table 1) to help trainees achieve competence in the management of osteoporosis and a number of other rare MBD by the end of their training. The choice of topics was based on the published curriculum [2], trainee feedback and the experience of one the authors (ASJ) who has been running the MBD module of the MSc (endocrinology) QMUL course over the past 16 years.

**Table 1.** Programme on metabolic bone diseases for Specialist Rheumatology Trainees

Title	Format	Duration in minutes
Unexpected DXA results (6 cases)	Interactive	30
Fracture risk assessment, FRAXplus^®^, with emphasis on type 2 diabetes and corticosteroids	Lecture	20
Three more clinical cases (osteoporosis in men, osteonecrosis of the jaw and atypical lateral fracture of the femur)	Interactive	30
Prevention of osteoporosis: non-pharmacological measures	Lecture	20
Calcium and vitamin D (& The VITamin D and OmegA-3 Trial (VITAL))	Lecture	20
UK clinical guideline for the prevention and treatment of osteoporosis 2022	Lecture	30
Ten clinical cases quiz (including Paget’s disease in bone, calcinosis cutis, circumscripta and universalis)	Interactive	30

During the first 30 min, 6 cases of unexpected DXA scan results were presented. They included artifactually normal results, discrepancy between bone mineral density (BMD) in the lumbar spine and the hip and others. This was followed by a lecture on fracture risk assessment, FRAXplus^®^, MBD in type 2 diabetes and corticosteroids induced osteoporosis. Three more cases were discussed addressing osteoporosis in men, osteonecrosis of the jaw and early detection of atypical fracture of the femur, rare but significant complications of antiresorptive therapy. Three lectures followed covering non pharmacological prevention of osteoporosis, calcium, vitamin D, the VITamin D and OmegA-3 Trial (VITAL)) and the 2022 UK clinical guidelines for the prevention and treatment of osteoporosis. In the final session, 10 clinical cases were presented and discussed covering Paget’s disease of bone, calcinosis, hypercalcemia and inherited bone diseases.

The feedback from the attendees was very positive, with 98% thought the event was relevant and the satisfaction rate for the individual sessions scoring 88 to 92%. A number of trainees requested attendance to our MBD clinic to gain confidence in assessing and managing patients. We intend to repeat the course within 2 years of the first but with more emphasis on interpreting DXA scans, better assessment of fracture risk, planning personalised long term management of patient with osteoporosis and other less common MBD.

To complement this session, we also held a whole-day training session at the Royal National Orthopaedic Hospital (RNOH). This included interactive case-based discussions around osteoporosis referrals for high-risk patients and individual lectures covering osteomalacia, Paget’s disease, osteogenesis imperfecta, raised alkaline phosphatase, hypophosphataemia and hypophosphatasia. Importantly, this session introduced trainees to specialists working at the RNOH and an open invitation was made for trainees to join specialist clinics at later dates. These meetings can act as a helpful gateway to gaining further experience and create opportunities for the trainee to pursue research in this area if they wish, which may well be relevant given Hawarden et al.’s encouraging finding that 20% of trainees were considering subspecialisation in MBD.

Whilst these organised sessions are a popular method of covering the vast subject of MBD, they do not address the issue found in the survey that 73% of trainees are struggling to gain hands-on experience of managing MBD patient to consolidate their learning. This is where the role of the MBD-lead could be most influential, helping to facilitate distribution of specialist BMD clinic experience throughout a region so that each trainee gains experience of 2 or 3 specialist clinics, whether they are able to rotate through a specialist centre or not.

Perhaps the most surprising finding from the survey is that by ST7, 56% of responders had not participated in osteoporosis clinic, a core topic for the general Rheumatologist. We read with interest that proposed barriers to attaining this experience included the move to the new General Internal Medicine (GIM) curriculum.

The 2022 Stage 2 internal medicine curriculum [3] requires each trainee to take part in a minimum of 20 outpatient clinics in other specialities, 'the choice of clinic should be driven by the educational needs of the trainee, as identified by the trainee and their educational supervisor’. Whilst osteoporosis is strictly not outside of the Rheumatology specialty, it is also shared by endocrinology and care of the elderly and this may be a part of the GIM curriculum that could work to serve a trainee’s educational needs in this area. This is not a substitute for the fact that the Rheumatology training layout should be meeting this educational need in the first instance, another area which could be addressed by introduction of a MBD lead.

We strongly support the recommendation by the authors that each region adopt a metabolic bone disease lead to oversee a systematic approach to training in order to meet curriculum requirements and maintain interest in the subspecialty in rheumatology, endocrinology and care of the elderly medicine.

## Data Availability

The data underlying this article will be shared on reasonable request to the corresponding author.
